# Psychometric properties of the parent-rated assessment scale of positive and negative parenting behavior (FPNE) in a German sample of school-aged children

**DOI:** 10.1186/s13034-024-00850-9

**Published:** 2024-12-16

**Authors:** Vanessa Holas, Ann-Kathrin Thöne, Christina Dose, Stephanie Gebauer, Christopher Hautmann, Anja Görtz-Dorten, Lea Teresa Kohl, Julia Plück, Anne-Katrin Treier, Tobias Banaschewski, Ulrike Ravens-Sieberer, Veit Rößner, Charlotte Hanisch, Michael Kölch, Martin Holtmann, Katja Becker, Tobias Renner, Julia Geissler, Jasmin Wenning, Michael Huss, Luise Poustka, Manfred Döpfner, the ADOPT, the ESCAlife consortia, Pascal-Maurice Aggensteiner, Dorothee Bernheim, Christian Beste, Stefanie Bienioschek, Maren Boecker, Daniel Brandeis, Kristina Butz, Andrea Daunke, Jörg M. Fegert, Claudia Ginsberg, Franziska Giller, Carolina Goldbeck, Martin Hellmich, Christine Igel, Michaela Junghänel, Anne Kaman, Anne Ritschel, Veit Roessner, Jennifer Schroth, Anne Schüller, Marion Steiner, Anne Uhlmann, Daniel Brandeis, Nina Christmann, Ute Dürrwächter, Lena Flick, Brigitta Gehring, Nicole Grau, Monja Groh, Wiebke Haberhausen, Johannes Hebebrand, Alisa Hiery, Sarah Hohmann, Christine Igel, Thomas Jans, Karina Jansone, Katja John, Anna Kaiser, Daria Kasperzack, Julia Kellner, Johanna Ketter, Franziska Kieninger, Inken Kirschbaum-Lesch, Katrin Krugmann, Tanja Legenbauer, Christopher Mann, Franziska Martin, Sabrina Millenet, Tanja Mingebach, Melinda Mross, Anja Pascher, Louisa Poustka, Marcel Romanos, Priska Schneider, Anja Schöllhorn, Bastian Schrott, Karen Schulze-Husmann, Thomas Stehr, Ida Steinacker, Marie-Therese Steiner, Ann-Katrin Thöne, Henrik Uebel-von Vandersleben, Paula Vetter, Linda Weber, Anne-Kathrin Wermter, Matthias Winkler, Elena Wirth, Mirjam Ziegler

**Affiliations:** 1 Department of Child and Adolescent Psychiatry, Psychosomatics and Psychotherapy, Faculty of Medicine and University Hospital Cologne, University of Cologne, Cologne, Germany; 2 School for Child and Adolescent Cognitive Behavior Therapy (AKiP), Faculty of Medicine and University Hospital Cologne, University of Cologne, Cologne, Germany; 3 Department of Child and Adolescent Psychiatry and Psychotherapy, Medical Faculty Mannheim, Central Institute of Mental Health, University of Heidelberg, Mannheim, Germany; 4 Department of Child and Adolescent Psychiatry, Psychotherapy, and Psychosomatics & Research Unit Child Public Health, University Medical Center Hamburg-Eppendorf, Hamburg, Germany; 5 Department of Child and Adolescent Psychiatry and Psychotherapy, TU Dresden, Dresden, Germany; 6 Faculty of Human Sciences, University of Cologne, Cologne, Germany; 7 Department of Child and Adolescent Psychiatry/ Psychotherapy, University of Ulm, Ulm, Germany; 8 Medical Center, Rostock University, Rostock, Germany; 9 German Center for Child and Adolescent Health (DZKJ), Partner Site Greifswald/Rostock, Site Rostock, Rostock, Germany; 10 LWL-University Hospital for Child and Adolescent Psychiatry, Ruhr-University Bochum, Hamm, Germany; 11 Department of Child and Adolescent Psychiatry, Psychosomatics and Psychotherapy, Medical Faculty of the Philipps-University Marburg and University Hospital Marburg, Marburg, Germany; 12 Department of Child and Adolescent Psychiatry and Psychotherapy, University Hospital Tübingen, Tübingen, Germany; 13 Center of Mental Health, Department of Child and Adolescent Psychiatry, Psychosomatics and Psychotherapy, University Hospital of Würzburg, Würzburg, Germany; 14 Department of Child and Adolescent Psychiatry, Psychosomatics and Psychotherapy, University Hospital Essen, University of Duisburg-Essen, Essen, Germany; 15 Department of Child and Adolescent Psychiatry and Psychotherapy, University Medical Center of the Johannes Gutenberg University Mainz, Mainz, Germany; 16 Department of Child and Adolescent Psychiatry, Center for Psychosocial Medicine, University Hospital Heidelberg, Heidelberg, Germany

**Keywords:** Parenting, Children and adolescents, Assessment, Psychometric properties, Externalizing disorders

## Abstract

**Background:**

The aim of this study was to develop and psychometrically evaluate a parent-rated parenting assessment scale including positive and negative dimensions of parenting. Factorial validity, reliability, measurement invariance, latent mean differences and construct validity of the Assessment Scale of Positive and Negative Parenting Behavior (FPNE) were tested in a pooled sample of five studies of 1,879 school-aged children (6.00 to 12.11 years).

**Methods:**

Exploratory factor analysis (EFA) was performed on a first randomized split-half sample, and confirmatory factor analysis (CFA) and exploratory structural equation modeling (ESEM) were conducted in the second half of the sample. Measurement invariance tests were conducted to assess factor structure equivalence across gender and age.

**Results:**

The EFA results supported a two-factor structure and the CFA results revealed a model with two correlated factors (Positive Parenting, Negative Parenting), which included 23 items and showed acceptable model fit and good psychometric properties. ESEM did not yield a model with significantly better model fit. Internal consistencies were acceptable. Adequate concurrent validity was demonstrated by low to moderate correlations between the FPNE and similar constructs. The factor structure was invariant (configural, metric, scalar) across different age groups and gender. Tests of latent mean differences revealed that older children scored significantly higher on negative parenting than younger children, while boys showed lower levels of positive parenting and higher levels of negative parenting compared to girls. All effect sizes were small.

**Conclusions:**

The results suggest that the FPNE is a reliable and valid instrument for the assessment of parenting.

**Supplementary Information:**

The online version contains supplementary material available at 10.1186/s13034-024-00850-9.

## Introduction

The importance of family factors in the development and maintenance of mental disorders in children and adolescents is one of the best studied topics in etiological research [1]. In particular, numerous studies have underlined the impact of parenting on psychopathological problems in children e. g. [2, 3]. When conceptualizing parenting, it is important to distinguish between attitude and behavior: Attitude refers to parents' internalized cognitions and beliefs about parenting, including parental goals and knowledge, whereas parental behavior describes the way of acting in certain situations. In the present paper, we focus on parental behavior, although it should be noted that an individual’s behavior is closely related to his or her attitudes [4].

Empirical research has yielded various different dimensions of parenting behaviors. Emotional responsiveness, for instance, refers to the degree to which parents behave in a supportive, accepting, nurturing, and warm manner towards their child [5]. Behavioral control comprises parental practices such as guiding, directing, setting limits and monitoring, in which children can recognize clear and consistent expectations, helping to regulate their behavior [6]. Another key aspect of parenting concerns the extent to which parents support their children’s autonomy (autonomy-granting), defined as the independence with which children are allowed to act and make decisions for themselves [7]. Psychological control refers to intrusive and manipulative parental behaviors that are specifically aimed at controlling the child and thereby exploit the emotional parent–child relationship [8]. Harsh control covers a range of highly destructive parenting practices including psychological and physical punishment, neglect, and intrusiveness [9, 10]. Emotional responsiveness, behavioral control, and autonomy-granting are described as functional parenting dimensions, which are associated with positive outcomes in child development and are thus termed positive parenting, whereas psychological control and harsh control are described as dysfunctional parenting dimensions, which are related to negative outcomes– so-called negative parenting [9, 10].

In view of the significant impact of these behaviors on child development, there has long been a strong interest in the development of parenting assessments [9, 10]. However, the majority of existing questionnaires have either insufficient psychometric properties or assess different aspects of parenting. Several measures focus exclusively on dysfunctional aspects, and only a small number of instruments assess both positive and negative parenting practices [11], despite research demonstrating the relevance of both dimensions with regard to effects on child outcomes [3]. In a review of the psychometrics of parenting measures, Hurley et al. [11] reported that only five out of 164 assessments showed acceptable psychometric properties. Of these, only the Alabama Parenting Questionnaire APQ, [12] captures both positive and negative aspects of parenting, with scales related to conduct problems and delinquency. The other four questionnaires are either very specifically oriented Parenting Alliance Measure [PAM], [13]; Parenting-Child Relationship Inventory [PRCI], [14] or assess parenting unidimensional Child Abuse Potential Inventory [CAPI], [15]; Parenting Scale [PS], [16]. The *Questions on Parenting* FZEV; German: Fragen zum Erziehungsverhalten; [17] was developed as part of the evaluation of the Triple-P program, a preventive parental intervention to reduce disorders in children. Based on several English-language questionnaires, including the Parenting Practices Scale by Strayhorn and Weidmann [18], the FZEV exclusively measures positively reinforcing and nurturing parenting behavior, and has been evaluated in several studies e.g., [19–21]. In 2017, Parent and Forehand published the Multidimensional Assessment of Parenting Scale (MAPS), a new instrument covering the two domains positive and negative parenting with seven subdimensions. Initial analyses in a U.S. sample provided promising evidence for its psychometric properties. The MAPS includes items from the APQ, the Parenting Scale, and the Management of Children’s Behavior Scale– Revised MCBS, [22, 23].

In line with children's development, some parenting practices also change over time [24]. Children exhibit different behaviors depending on their age and gender, which can elicit different reactions from parents [3, 25]. However, the majority of parenting measures do not appear to reflect this development, with the exception of the MAPS. To facilitate research and clinical work, a time-efficient assessment of parenting with established measurement invariance across child gender and age based on well-evaluated instruments is imperative. Despite the substantial number of existing instruments, to date, there are no measures that assess parenting in a very simple, structured, and economical manner.

The present study therefore aimed to develop and evaluate a brief and comprehensive measure of parenting, including both positive and negative parenting practices, that meets psychometric standards with established measurement invariance to facilitate the assessment of parenting behaviors in clinical and research settings.

## Method

### Participants

The overall sample of this study comprised 1,879 children (aged 6 to 12 years), mostly with symptoms or diagnoses of externalizing behavior disorders. Data were collected within the scope of five studies on the treatment of externalizing behaviors, which were coordinated by the Department of Child and Adolescent Psychiatry, Psychosomatics and Psychotherapy at the University Hospital of Cologne, Germany: (1) ADOPT study—Affective Dysregulation—Optimizing Prevention and Treatment [26], (2) Self-Help Comparison Study—Behavioral Versus Nonbehavioral Guided Self-Help for Parents of Children with Externalizing Disorders [27], (3) Enhancement Study—Telephone-Assisted Self-Help for Parents of Children with Attention-Deficit/Hyperactivity Disorder who have Residual Functional Impairment despite Methylphenidate Treatment [28], (4) ESCAschool Study—Evidence-Based Stepped Care of ADHD [29], and (5) WASH Study—Efficacy of Web-Assisted Self-Help for Parents of Children with ADHD [30]. From the research consortium ADOPT, three subprojects provided data for this study: ADOPT Online, ADOPT Treatment, and ADOPT Institution. ESCAschool is a sub-study within the research consortium ESCA-life. Across all subprojects, caregiver ratings of school-age children (6.00 to 12.11) were included.

The majority of the participants had a suspected or confirmed diagnosis of attention-deficit/hyperactivity disorder (ADHD), oppositional defiant disorder (ODD), hyperkinetic disorder (HKD), and/or current symptoms of ADHD/ODD/HKD or affective dysregulation (AD). Across the studies, inclusion criteria were no severe cognitive impairment in the children, and willingness for study participation of a parent. In the ADOPT and ESCA study, children's informed consent was also obtained. Exclusion criteria included severe intellectual disability (e.g., IQ < 80), pervasive developmental disorder, another primary disorder (e.g., autism spectrum disorder), or severe psychiatric conditions such as schizophrenia, bipolar disorder, or major depressive episode. Children undergoing current or planned behavioral therapy or indication for inpatient treatment. The samples were recruited Germany-wide through facilities of the local health care systems, residents’ registration offices, and out-of-home care institutions.

Data of 1,879 parents of children aged between 6.00 and 12.11 years were included (*n*_ADOPT_ = 695; *n*_Self-help comparison_ = 104; *n*_Enhancement_ = 114; *n*_ESCA_ = 559; n_WASH_ = 407). Children who were still attending kindergarten were excluded from the data set of the self-help comparison study (*n* = 45).

### Measures

The *Assessment Scale of Positive and Negative Parenting Behavior (FPNE—Fragebogen zum positiven und negativen Erziehungsverhalten)* was constructed based on items of established parenting scales and self-constructed items, which were selected by a focus group of experts in behavioral child and adolescent psychotherapy. We used all 13 items of the German *Questions on Parenting* (FZEV; 17), which assesses parenting behavior on a four-point rating scale (0 = *never* to 3 = *very often*), with higher scores indicating more positive reported parenting behavior. The FZEV is a German adaptation of the Parent Practices Scale PPS; [17, 18], and was developed and used to evaluate a Triple-P parenting training intervention and a prevention program for externalizing problem behavior. Internal consistencies of the total scale ranged from α = 0.84 to α = 0.87 [30, 31]; α = 0.86 in the present study. Additionally, 13 items were selected from the *Management of Children’s Behavior Scale– Revised* MCBS, [23], which were partly modified during the translation process into German. The original version of the MCBS consists of 37 items rated on a three-point Likert scale (1 = *not like me*, 2 = *somewhat like me*, and 3 = *like me*), with higher scores indicating stronger identification with the stated parenting behavior. The scale covers coercive communication, acknowledgment of good behavior, physical punishment, harsh punishment, inconsistent parental control, and negative reinforcement of deviant behavior. From each category, we selected one to five items which demonstrated a part-whole-corrected item-total correlation of *r*_it_ ≥ 0.30 in the analysis by Perepletchikova and Kazdin [23] and which lent themselves well to translation into German. The original 37-item version of the MCBS has shown an internal consistency of α = 0.84 [23]. Furthermore, a focus group of therapy experts created sixteen additional self-constructed items relating to dysfunctional parenting (example: “Whether or not I impose a punishment often depends on my mood.”). As the response format for the FPNE, we chose the four-point rating scale (1 = never to 4 = very often) used in the FZEV. For the items adopted from the MCBS scale and the self-constructed items, the scale was adjusted accordingly. A detailed overview of the items and their origin can be found in Table S1 of the supplement.

The following instruments were used to assess the validity of the newly constructed scale: The *Symptom Checklists for Attention-Deficit/Hyperactivity Disorder (FBB-ADHS;* German: “Fremdbeurteilungsbogen für Aufmerksamkeitsdefizit-/Hyperaktivitätsstörungen”, 20 items, [32]*)* and *for Oppositional Defiant Disorder and Conduct Disorder (FBB-SSV;* German: “Fremdbeurteilungsbogen für Störungen des Sozialverhaltens”, 24 items, [32]*)* assess ADHD and ODD/conduct disorder symptoms according to the DSM-5 and ICD-10. The ADHD total scale and the ODD scale were used in the present study. Both scales have shown satisfactory internal consistency and factorial validity (ADHD Total, 20 items, α = 0.94; ODD Total, 8 items, α = 0.87; [33]). Internal consistencies in the present study were α = 0.95 (ADHD Total) and α = 0.81 (ODD Total). All items were rated on a four-point Likert-type scale ranging from 0 to 3 (0 = *not at all* to 3 = *very much*), with higher scores indicating higher symptom severity. Both instruments are part of the Diagnostic System for Mental Disorders in Childhood and Adolescence DISYPS-III; [32]. For studies conducted with the previous version of the DISYPS– the DISYPS-II [34]– the data were combined.

From the German translation of the *Child Behavior Checklist for Ages 6–18* CBCL 6-18R, 113 items, [35], we used the symptom scales to assess externalizing symptoms (CBCL External, 35 items) and internalizing symptoms (CBCL Internal, 32 items). Each item was rated on three-point Likert scale (0 = *not true* to 2 = *true*). Scale scores were calculated by summing the individual item scores, with higher scores indicating more severe behavior problems. Both second-order scales have shown satisfactory validity and internal consistencies above 0.80 (CBCL External α = 0.93; CBCL Internal α = 0.86; 34). In the present study, internal consistencies were α = 0.90 (CBCL External) and α = 0.84 (CBCL Internal).

The *Depression Anxiety Stress Scales* (DASS-21; 36; German: Depressions-Angst-Stress-Skalen, 21 items) measure symptoms of depression, anxiety, and stress referring to the previous week. We employed a German adaptation of the 42-item version by Lovibond and Lovibond [36]. The items were rated on a four-point Likert scale (0 = *never* to 3 = *very often*), with higher scores indicating higher symptom severity. The questionnaire consists of the three scales Depression (7 items; α = 0.88), Anxiety (7 items; α = 0.76), and Stress (7 items; α = 0.86; 36), which have shown satisfactory internal consistencies and factorial validity [37]. In the present study, internal consistencies were α = 0.86 (Depression), α = 0.73 (Anxiety), and α = 0.82 (Stress).

The *KIDSCREEN-10 *10 items [38] assesses various aspects of health-related quality of life and well-being in children and adolescents. The total quality of life score was used in the present study (KIDSCREEN Total, 10 items). The items were rated on a five-point Likert scale (0 = *never/not at all* to 4 = *very often*), with higher scores indicating higher quality of life and/or well-being. Internal consistency for the total scale was satisfactory, at α = 0.82, and factorial validity was demonstrated [38]. In the present study, internal consistency was α = 0.77.

### Data analyses

All statistical analyses were performed using SPSS 28, R 4.4.0 or Mplus. Descriptive, reliability, and validity analyses were conducted separately in the total sample and in the subsamples originating from the different included studies.

An analysis of missing data in the total sample revealed 2.6% missing values for items of the FPNE across all caregivers. In cases of less than 10% missing values in the questionnaire per caregiver, they were conservatively replaced by 1 ("behavior is not shown"; *n* = 5). Questionnaires with more than 10% missing values were excluded from the analysis (*n* = 4).

For initial item selection and scale development of the FPNE, we performed exploratory factor analyses (EFA). To determine the best-fitting model, we estimated and sequentially compared two two-factor CFA models (first-order correlated-factors model, modified first-order correlated-factors model) and used exploratory structural equation modeling (ESEM). Validation of the factor structure was undertaken by randomly dividing the total sample into two halves, performing the EFA in the first subsample and the CFA and ESEM in the second subsample.

In psychological research, ordinal data often studied using factor analysis with Pearson correlation, constructed for metric and normally distributed data e.g., [3]. Our analyses of skewness and kurtosis, as well as our tests for normal distribution, showed significant deviations from the normal distribution for several items. The use of a polychoric correlation matrix is actually the more reliable method to create a more accurate measurement model that better reflects the latent relationships between items [39]. After careful consideration, we decided to combine both methods. To ensure methodological comparability with other studies, an exploratory factor analysis (principal axis analysis using oblimin rotation) based on Pearson correlations was first performed in SPSS initially without specifying the number of factors and second with specification of 2, 3, and 4 factors. The two-factor model was based on the assumption that there could be a global positive and a negative factor. To determine the appropriate number of factors to retain in the exploratory factor analysis (EFA), we used several criteria: the Kaiser criterion (eigenvalues > 1), the MAP test, and parallel analysis. We also chose a threshold for factor loadings of ≥|.32|, which is a commonly used threshold in factor analysis, to ensure that the item contributions to the factors are significant [40]. The two-factor model was then tested and compared in R using the psych and lavaan packages based on polychoric correlations.

For confirmatory factor analysis, we used the robust weighted least squares with mean and variance adjustment (WLSMV) estimator, as it does not assume normally distributed data [41]. To evaluate the model fit, the comparative fit index (CFI), the Tucker–Lewis index (TLI), the root mean square error of approximation (RMSEA) with corresponding 90% confidence intervals, and the standardized root mean square residual (SRMR) were considered as goodness-of-fit indices [42]. Given its dependence on the sample size, less attention was paid to the χ^2^ test. An RMSEA and SRMR ≤ 0.08 and CFI/TLI values > 0.90 were considered to be indicative of acceptable model fit. CFI/TLI values > 0.95 and RMSEA and SRMR values ≤ 0.05 were considered to indicate good model fit [42, 43]. Regarding the CFA, in the first-order correlated-factors model, correlations between the positive and the negative parenting factor were allowed. In contrast to the CFA models, the ESEM permitted cross-loadings between all items to prevent overestimation of factor loadings.

Additionally, we tested the measurement invariance of the final model for children’s age (6.00 to 9.56 vs. 9.57 to 12.11) and gender (male vs. female). Based on the median, the children were divided into two groups (*Mdn* = 9.56). For this purpose, stepwise restrictions were performed by constraining parameters to be equal across the groups. Strong factorial invariance is given when 1) configural, 2) metric, and 3) scalar invariance is achieved [44]. Restrictiveness increases with the different types of measurement invariance [41]. For configural measurement invariance, the same model was estimated in both groups, and the factor loadings, intercepts, and residual variances were allowed to vary freely. Configural measurement invariance was given in the case of the same loading pattern and number of factors. Metric invariance means that all groups have the same conceptual understanding of the latent constructs. For the examination of metric invariance, the loadings are additionally set to be equal in all groups. Scalar invariance means that the item difficulties do not differ between the groups. Additionally, the intercepts of the manifest variables in all groups have to be the same [45]. As the difference test is sample-sensitive, Chen's [46] rule of thumb was used to assess the model comparisons. Accordingly, the CFI should not decrease by more than 0.02 and the RMSEA should not increase by more than 0.015.

Following the testing for measurement invariance, latent mean differences between age and gender groups were examined, starting from the full scalar invariance model. To compare differences in latent means between groups, the mean for younger children and girls was set to zero as the reference group, while the means for older children and boys were estimated freely. This approach does not estimate absolute means for each group but rather assesses mean differences in the latent variables between the groups. Latent mean differences were evaluated using the critical ratio (CR) index, where CR values of 1.96 or above indicate significant differences. A positive CR value suggests that the comparison group has higher latent means than the reference group. Effect sizes were evaluated using Cohen’s *d*, which expresses the group mean difference as a proportion of the pooled within-group standard deviation. Following Cohen’s guidelines [47], we used the following interpretations for *d*: 0.20 ≤ *d* ≤ 0.39 as small, 0.40 ≤ *d* ≤ 0.79 as moderate, *d* ≤ 0.80 as large.

Subsequently, internal consistency (Omega, Cronbach’s alpha) and concurrent validity of the final model were examined. To assess validity, correlations between scale scores and parent-reported child ADHD/HKD/ODD symptoms (FBB-ADHS, FBB-SSV), child externalizing or internalizing symptoms (CBCL/6-18R), children’s quality of life (KIDSCREEN-10), and parental psychological symptoms (DASS-21) were examined using Pearson correlation coefficients (*r*). Based on the results of Pinquart [9, 10], we hypothesized that negative parenting would show a small to moderate association with psychopathological symptoms of children and parents; in relation to quality of life, we expected negative low to medium correlations. With regard to positive parenting behavior, we expected negative correlations in the low range with psychological problems of children and parents, and a small to moderate positive correlation with quality of life, as found in previous studies (e.g., 9, 10).

## Results

### Sample characteristics

Characteristics of the overall sample are displayed in Tables 1 and 2 (for characteristics of the subsamples, see Table S2 of the supplement). In the overall sample, the majority of the children were male (73.4%) and the average age was 9.70 (*SD* = 1.64) years. Overall, 13.5% of the children had special educational needs. Most of the children had a diagnosis either of ADHD, ODD including HKD, or symptoms of affective dysregulation (77.6%). The rest of the sample showed no symptoms (22.4%). Participating caregivers had an average age of 41.40 years (*SD* = 6.32), the majority reported their country of origin as Germany (87.9%), and 17.2% were single parents. Probably due to the large total sample size, we found significant differences in demographic characteristics between participants from different studies in all analyses. However, only the differences in age of the child (*d* = 1.02), any diagnosis (*V* = 0.59), medication (*V* = 0.89) and single-parent status (*V* = 0.52) were meaningful with regard to the effect sizes. These differences can be explained by the different inclusion criteria of the studies.

**Table 1:** Child demographic characteristics for total sample

	*N*	Age (years): *M* (*SD*) [range]	Gender (male)			Type of school			Special educational needs (yes)	Diagnosis ADHD/ODD incl. HKD (yes)	ADHD medication (yes)
				Primary school	Special school	Secondary school	High school	Other school			
Total sample	1879	9.70 (1.64) [7.48]	73.4%	61.7%	7.3%	11.3%	19.5%	1.6%	13.5%	77.6%	32.4%

M = mean, SD = standard deviation, ADHD = Attention Deficit Hyperactivity Disorder, ODD = Oppositional Defiant Disorder, HKD = Hyperkinetic Disorder, Special educational needs defined as “a learning difficulty which calls for special educational provision to be made” [49, p. 6]

**Table 2:** Parent demographic characteristics for total sample

	*N*	Age participant (years): *M (SD)* [range]	Single-parent status(yes)	Country of origin (Germany)	Language spoken at home (German)
Total sample	1879	41.40 (6.32) [42.77]	17.2%	87.9%	96.0%

M = mean, SD = standard deviation

### Exploratory factor analysis

A preliminary analysis of the FPNE instrument was conducted by Imort et al. in 2014 [48], based on data from 146 parent ratings. Since then, the 38-item instrument has been used in several larger studies at the Department of Child and Adolescent Psychiatry, Psychosomatics and Psychotherapy at the University Hospital of Cologne. These studies provided a more comprehensive dataset, which forms the basis for the current psychometric analysis. Given the larger and more representative sample now available, we have focused the present analysis on these data. Supplementary Table S3 shows the EFA results for the initial 38-point solution of this study.

Results of the final EFA can be found in Table 3. Velicer’s minimum average partial (MAP) test and a parallel analysis supported a two-factor structure see Supplement Figs. F1 to F4, [50]. We excluded six items due to standardized factor loadings below the cut-off value of 0.32 and four items with factor loadings that could not be interpreted (e.g., an item which addresses positive parenting strategies showed positive cross-loadings on factor 2, where only items addressing negative parenting strategies showed positive loadings), resulting in 17 items loading on factor 1 and 11 items loading on factor 2. To make the instrument more economical, factor 1 was additionally reduced to the 13 items of the FZEV scale [17]. The Cronbach’s Alpha for the original scale with 17 items was 0.89, while the final scale with 13 items yielded an Alpha of 0.86, indicating that the exclusion of the other four items did not significantly reduce internal consistency (see supplement Table S4for more details). On factor 2, the item 12 ("I often disagree with my partner (or significant other) about whether or not a particular behavior of my child should be punished") was dropped, as single parents did not complete it despite the reference to “significant other”. The final version of the FPNE consisted of 23 items in total, with 13 items of factor 1 (0.35 ≤ *r* ≤ 0.71) and 10 items of factor 2 (0.34 ≤ *r* ≤ 70). For item statistics, item-level correlations, scale statistics, and test statistics of the FPNE, see supplement Table S5. Based on the Pearson matrix correlation, a total of 32.34% of the variance was explained (*R*^2^ factor 1 = 22.54%; *R*^2^ factor 2 = 9.81%). Based on the polychoric matrix correlation, a total of 41.05% of the variance was explained (*R*^2^ factor 1 = 25.47%; *R*^2^ factor 2 = 15.58%). Loadings based on polychoric correlations (PP: 0.39 ≤ *r* ≤ 0.78; NP: 0.33 ≤ *r* ≤ 0.77) are consistently higher than loadings based on Pearson correlations (PP: 0.33 ≤ *r* ≤ 0.70; NP: 0.26 ≤ *r* ≤ 0.72). As mentioned, polychoric correlations are considered more meaningful for ordinal data. The influence is particularly evident in item 09, which does not exceed the cut-off value of 0.32 based on Pearson correlations, but is based on polychoric correlations and is therefore retained in the final version.

**Table 3:** Final exploratory factor analysis results with specification of 2 factors (N = 917)

	Factorial loads based on Pearson correlation matrix		Factorial loads based on polychoric correlation matrix	
	PP	NP	PP	NP
17. I say something nice to my child	**.70**		**.78**	
29. I laugh with my child	**.69**		**.77**	
4. I have fun with my child	**.68**		**.76**	
32. I praise my child	**.66**		**.76**	
25. I cuddle with my child	**.61**	-.22	**.66**	-.24
26. I do things with my child	**.61**		**.67**	
2. I talk to my child	**.60**		**.72**	
33. I tell my child things about myself	**.60**		**.66**	
24. I play with my child	**.54**		**.63**	
1. I show my child appreciation when he does things I like	**.52**	-.14	**.62**	-.16
34. If my child wants to show me something, I take the time for it	**.49**	-.17	**.58**	-.19
37. If my child comes to me and I’m busy, I try to include him/her in my activity	**.33**	-.10	**.38**	-.11
31. I do role-plays or puppet shows with my child	**.33**		**.39**	
18. I threaten to punish my child for his misbehavior, but I do not follow through		**.72**		**.77**
8. Whether or not I impose a punishment often depends on my mood		**.70**		**.75**
5. I punish my child for doing something one day, but ignore it the next day		**.63**		**.71**
27. I take away a privilege but if my child whines or complains, I will give it back		**.56**		**.63**
22. I notice in myself that I talk insistently at my child in stressful or conflict situations	-.10	**.51**	-.11	**.54**
7. Whenever I ask my child to do certain things, I discuss it with him/her for a long time		**.51**		**.54**
19. If my child misbehaves, I will swear at him or call him names	-.13	**.45**	-.14	**.48**
6. I’m often irritated if my child wants to play with me	-.19	**.40**	-.22	**.45**
36. ‘If my child does something I’ve asked, I sometimes say: ‘Why don’t you always do that’?		**.35**		**.38**
9. I believe that if my child had misbehaved during the day, none of his good behavior should be rewarded	-.17	**.26**	-.21	**.33**

In bold, the factor loadings higher.32 (*p* <.05)

Principal axis factor analysis with oblimin rotation. PP = Positive Parenting. NP = Negative Parenting. *R*^2^ = 32.34 based on Pearson correlation matrix. *R*^2^ = 41.05 based on polychoric correlation matrix

### Confirmatory factor analysis

The CFA were performed based on the final exploratory model as described above, but in the other split-half sample. Goodness-of-fit indices of the first-order correlated-factors model (Model 1) indicated an acceptable model fit (CFI = 0.920, TLI = 0.911, RMSEA = 0.066, SRMR = 0.064). Factor 1 and factor 2 correlated at *r* = -0.41. In the modified first-order correlated-factors model (Model 2), two item residual covariances were additionally considered appropriate and allowed (Item 01 “I show my child appreciation when he does things I like.” with Item 19 “I praise my child.”; Item 13 “I play with my child.” with Item 15 “I do things with my child.”).The model fit for Model 2 was also acceptable, but slightly better than for Model 1: the CFI was 0.930, the TLI was 0.922, the RSMEA was 0.062 and the SRMR was 0.061. All standardized factor loadings were significant, with 0.41 ≤ *λ* ≤ 0.80 on factor 1 and 0.38 ≤ *λ* ≤ 0.73 on factor 2.

### Exploratory structural equation modeling

**Fig. 1:**
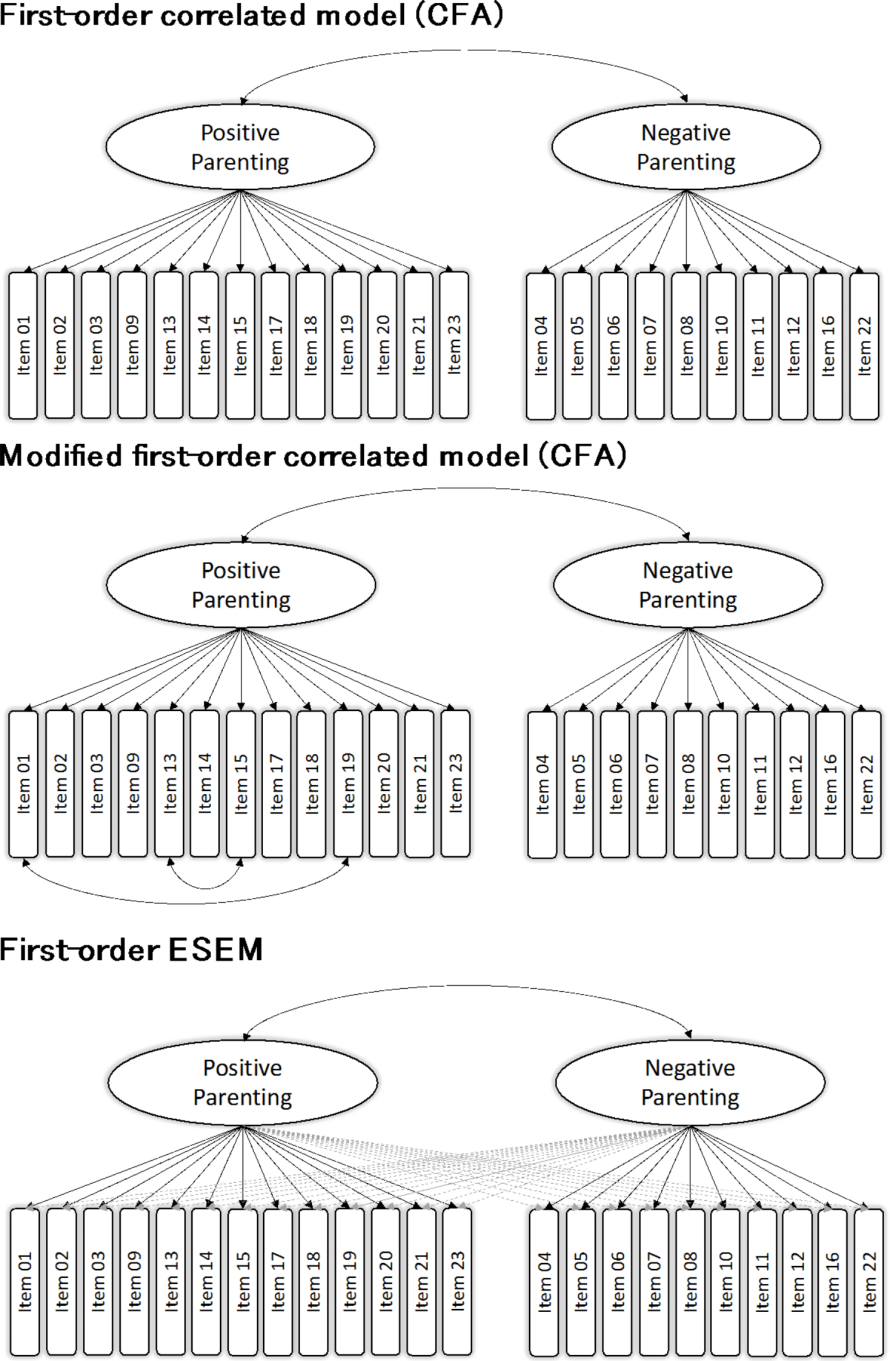
Factor models of parenting. Graphical overview of the factor models evaluated in this study. Full unidirectional black arrows indicate target factor loadings, while dashed unidirectional grey arrows indicate cross-loadings. Item numbers are shown within the boxes and residuals are not shown to improve clarity. CFA refers to confirmatory factor analysis and ESEM to exploratory structural equation modelling

The ESEM two-factor model also showed an acceptable model fit (CFI = 0.934, TLI = 0.920, RMSEA = 0.063, SRMR = 0.046). The factor loadings ranged between 0.39 ≤ *λ* ≤ 0.80 on factor 1 and 0.38 ≤ λ ≤ 0.80 on factor 2. The factor correlation was *r* = -0.35 (for an illustration of the three models, see Fig. 1). All cross-loadings of the items on all factors were below the limit of λ > 0.32 set by Tabachnick and Fidell [40]. Morin, Arens, and Marsh [51] suggest the use of the ESEM model when the model fit of the ESEM model is substantially better than that of the associated CFA model and if there are lower factor correlations. The ESEM model did not fit the data significantly better than the CFA Model 2 with regard to most indices (Δ*χ* 2 = − 67.229; Δ*df* = 19; ΔCFI = 0.004; ΔTLI = -0.002; ΔRMSEA = − 0.001; ΔSRMR = − 0.015). Therefore, the more restrictive CFA first-order correlated-factors model was retained.

### Measurement invariance

We analyzed measurement invariance across gender (male vs. female) and age groups (6.00 to 9.56 vs. 9.57 to 12.11). All fit indices are shown in Table 4. With free estimation of parameters, the configural measurement invariance is given across both gender and age groups. The constraining of the factor loadings did not decrease model fit for the factors gender and age (male vs. female and 6.00 to 9.56 vs. 9.57 to 12.11). The FPNE scales thus have the same meaning for children of different gender and age groups. Constraining the intercepts to be equal also did not lead to a decrease in model fit; thus, the scalar measurement invariance was confirmed and the items of the scales function equally for both age groups and both genders.

**Table 4:** Model fit indices of the invariance tests for the total sample (split-half)

		χ^2^ (*df*)	CFI	TLI	RMSEA [90% CI]	SRMR
Age (6.00 to 9.00 vs. 9.01 to 12.11)	Configural	1468.027* (475)	.915	.909	.066 [.062, .070]	.071
	Metric	1396.216* (496)	.923	.921	.062 [.058, .065]	.074
	Scalar	1470.208* (540)	.920	.925	.060 [.056, .064]	.074
Gender (female vs. male)	Configural	1353.887* (474)	.920	.915	.063 [.059, .066]	.069
	Metric	1234.009* (495)	.933	.932	.056 [.052, .060]	.071
	Scalar	1268.894* (539)	.934	.938	.053 [.050, .057]	.071

X^2^ = Chi-square, df = degrees of freedom, CFI = Comparative Fit Index, TLI = Tucker-Lewis Index, RMSEA = root mean square error of approximation, CI = confidence interval, SRMR = standardized root mean square residual, *N*_total_ = 917

*p <.05

### Latent mean differences

Based on the establishment of the full scalar invariance across both age and gender groups, we compared the latent mean differences across these groups for positive parenting and negative parenting. The younger children group and the female children group is selected as reference group and its latent mean is fixed to zero. Findings of the latent mean comparisons between ages of children and adolescents showed that older children had no significant different score than younger children on positive parenting (*E*_*st*_ = − 0.109 ± 0.073, CR = -1.494, *p* = 0.135, Cohen’s *d* = -0.12). For negative parenting, older children showed significantly higher scores than younger children (*E*_*st*_ = − 0.174 ± 0.071, CR = -2.440, *p* < 0.05, Cohen’s *d* = -0.17). Findings between gender of children and adolescents showed that boys had significant lower scores than girls on positive parenting (*E*_*st*_ = 0.223 ± 0.088, CR = -2.740, *p* < 0.05, Cohen’s *d* = -0.21) and significant higher scores for negative parenting (*E*_*st*_ = − 0.174 ± 0.071, CR = 2.544, *p* < 0.05, Cohen’s *d* = 0.24). However, the effect size for all mean differences was regarded as small.

### Reliability

Omega and Cronbach's alpha generally show a similar trend regarding the internal consistency of the FPNE scales. All three values confirm that the Positive Parenting Scale (ω = 0.87, α = 0.86) has a higher reliability than the Negative Parenting Scale (ω = 0.78, α = 0.78). Item-scale correlations were all moderate to high (PP *r*_it_ = 0.33 to 0.65; NP *r*_it_ = 0.31 to 0.58). Detailed information on the total sample and subsamples can be found in Table S6 of the supplement.

### Concurrent validity

The correlations of the two FPNE scales Positive Parenting (PP) and Negative Parenting (NP) were moderate in the complete sample (*r* = -0.32) and small to moderate in the subsamples (-0.39 ≤ *r* ≤ -0.23) (see Table S7 in the supplement). Associations between the two FPNE scales and similar constructs are presented in Table 5. While the Positive Parenting scale showed small negative correlations with ODD, ADHD, and externalizing symptoms (-0.26 ≤ *r* ≤ -0.17) and no significant correlation with internalizing symptoms, the Negative Parenting scale showed small to moderate correlations with ODD, ADHD, externalizing symptoms, and internalizing symptoms (0.18 ≤ *r* ≤ 0.35). A similar finding emerged for parental psychopathology, with a small negative correlation with the Positive Parenting scale (*r* = -0.18) and a moderate correlation with the Negative Parenting scale (*r* = 0.36). Additionally, we found a small correlation of both scales with the child’s global quality of life (NP *r* = -0.19; PP *r* = 0.26).

**Table 5:** Correlations of positive parenting (PP) and negative parenting (NP) Scale with children’s ODD symptoms, ADHD symptoms, internalizing and externalizing problems, and quality of life, and with parents’ anxiety, depression, and stress

	PP	NP	*n*
Child-related variables			
FBB-SSV			
ODD Total	-.26*	.35*	1649
FBB-ADHS			
ADHD Total	-.19*	.34*	1426
CBCL			
Internalizing problems	-.05	.19*	1063
Externalizing problems	-.17*	.29*	1063
KIDSCREEN-10			
Quality of life	.26*	-.19*	1195
parent-related variables			
DASS-21			
Depression	-.17*	.30*	1067
Anxiety	-.09*	.29*	1068
Stress	-.19*	.36*	1068

FBB-SSV = Symptom Checklists for Oppositional Defiant Disorder and Conduct Disorder (German: “Fremdbeurteilungsbogen für Störungen des Sozialverhaltens”), FBB-ADHS = Symptom Checklists for Attention-Deficit/Hyperactivity Disorder (German: “Fremdbeurteilungsbogen für Aufmerksamkeitsdefizit-/Hyperaktivitätsstörungen”), CBCL = Child Behavior Checklist for Ages 6–18, DASS-21 = *Depression Anxiety Stress Scales*

*p <.05

## Discussion

The aim of the current study was to develop and evaluate a brief and comprehensive assessment scale of parenting that encompasses positive and negative dimensions and is easy to use in clinical practice and research settings. The Assessment Scale of Positive and Negative Parenting Behavior (FPNE) was examined using an EFA, CFA, and ESEM. The final version of the FPNE contained 23 items and demonstrated an acceptable model fit and good item characteristics. The factor structure was invariant for children of different age and gender, whereas small significant latent mean differences were found. Furthermore, we found good internal consistencies and concurrent validity.

The EFA yielded a two-factor solution that explains a large proportion of the variance based on polychoric correlation matrix (41.05%) according to Cohen [47]. Consistent with the results of Holgado-Tello et al. [39], we found comparatively higher correlation measures and a larger proportion of explained variance using polychoric correlations than with Pearson correlations for ordinal data. Even the results of the CFA that permitted a correlation between the positive factor and the negative factor as well as the ESEM indicated an acceptable model fit. However, since the ESEM model did not provide a significantly better fit than the more restrictive CFA Model 2, we decided to retain the CFA model. It shows that the items measuring positive and negative parenting behaviors are very clear and selective for the respective factors. This indicates that cross-loadings are not necessary, as the items designed to measure positive parenting do not simultaneously capture aspects of negative parenting (and vice versa). Practically speaking, positive and negative parenting can be measured as separate and independent constructs without the items overlapping across both dimensions. For example, the item “I praise my child.” is clearly associated with positive parenting and does not significantly load on the negative factor. This distinction can be attributed to decades of research differentiating which parenting behaviors are clearly protective factors for child development and which are considered risk factors [9, 10]. The moderate negative correlation between the two factors suggests that parents who frequently engage in positive parenting behaviors (e.g., praise and recognition) tend to show less negative behavior (e.g., punishment or threats). However, it also indicates that these two behaviors are not completely oppositional but rather represent independent dimensions. Some parents may exhibit both types of behaviors (e.g., praising their child while also using punitive measures).

The Positive Parenting factor of the FPNE included items describing proactive parenting and the expression of emotional responsiveness, characterized by a warm and supportive parent–child interaction. The Negative Parenting scale reflected high levels of verbal hostility, laxness, inconsistent discipline, and harsh parenting. These behaviors correspond to the previously reported domains Emotional Responsiveness, Harsh Control, and Psychological Control e.g., [9, 10]. Both FPNE scales address various dimensions of parenting on an item basis, as they are also included in other psychometrically well-evaluated instruments e.g., MAPS; [3].

Invariance tests yielded satisfactory results across gender and age groups. The response behavior proved to be similar across gender and age groups, and it can thus be concluded that the FPNE measures the same construct across child development stages (age) and gender. In other words, parents of children between 6 and 12 years rate their parenting behaviors similarly, independently of their gender. Thus, the FPNE allows for comparisons of parenting domains across developmental stages in elementary school age children. As such, the instrument can also be used in future research to map the development of parenting and its effects on child development cf. [3] as well as to test the effects of parent training on parenting behavior.

Our results of tests of latent mean differences show that older children and boys had higher scores for negative parenting than younger children, while there was no significant difference between age groups for positive parenting. Boys had significantly lower positive parenting scores than girls. This could be due to the fact that externalizing behaviors are more common in adolescence and in boys, which could lead parents to respond with controlling or negative parenting methods [9, 52]. The lower levels of positive parenting among boys may indicate that parents provide less positive behaviors, such as affection or support, when they experience more externalizing behaviors. One possible explanation for this is that externalizing behaviors place greater demands on parents' patience, which may limit positive parenting. It is also possible that gender expectations play a role, with boys being perceived as more independent or less emotional, leading to less positive interaction. In practice, these effects could have implications for the design of more specifically parenting interventions.

Our findings that positive and negative parenting exert a small to moderate influence on externalizing symptoms, while negative parenting is associated with internalizing symptoms to a small but significant extent further support these conclusions. Nevertheless, no significant correlation was identified between positive parenting and internalizing behavior. These findings align with the results of Pinquart’s meta-analysis, which indicated a marginally stronger association between parenting and children’s externalizing behavior [9, 10]. Moreover, the evidence for direct effects of parenting on internalizing behavior has been inconsistent and limited in previous studies [9, 53–55]. One potential explanation for this discrepancy is the composition of the sample. Prior studies with a larger proportion of female participants have demonstrated more pronounced associations between positive parenting and the prevention of internalizing behaviors. Girls are more likely to exhibit internalizing symptoms, anxiety, and depression, and they often express their emotions more openly than boys [56]. Our study had a significantly higher proportion of boys mainly with externalizing problem behaviors, which could have further influenced the effect of positive parenting on internalizing behaviors. Meta-analyses have also identified a bidirectional relationship in which parents' behavior influences children's mental health, while children's behavior may also reinforce or modify parents' behavior in an escalating manner [9, 10]. As mentioned above, it is therefore possible that parents are more likely to engage in undesirable practices when children display externalizing behaviors. Children's externalizing behaviors may also push parents to their limits, leading to feelings of helplessness and increasing the likelihood of negative/less positive parenting behaviors [10]. Furthermore, the role of mediating factors in the effect of parenting on child behavior has been emphasized in a substantial body of research. Parenting may act indirectly through mechanisms involving genes, temperament or emotion regulation skills [53]. Consequently, future research should specifically examine how parenting affects child symptoms through other factors in order to gain a more comprehensive understanding of the mechanisms of action and potential intervention options. It is important to note that the inconsistent findings in previous research have not yet elucidated which dimensions of parenting are most strongly associated with problem behaviors in childhood and adolescence. With regard to internalizing symptoms, the evidence suggests that parental warmth and behavioral control are the most significant predictors [9]. Conversely, in the context of externalizing symptoms, harsh parenting and psychological control appear to exert the strongest influence [10]. Collectively, these findings indicate that multiple parenting dimensions may have an additive effect on child development, with a combination of parenting dimensions explaining a greater proportion of the variance.

Our results revealed that the FPNE scales for Positive (PP) and Negative (NP) Parenting show moderate to small correlations with externalizing symptoms, ADHD, ODD, child quality of life/well-being, and parental mental health in the overall and subsample analyses. These findings support the concurrent validity of the FPNE scales by demonstrating that they capture similar constructs and maintain consistent relations to related psychological variables. Prior research suggest that warm and supportive parenting behavior seems to be positively related to quality of life, while harsh or inconsistent parenting behavior seems to be associated with lower child well-being e.g., [57]. The evidence-based model developed by Newland [58] also assumes a pathway from parenting to child well-being, with parenting also seeming to play a mediating role in the association between family well-being and child well-being. Furthermore, a moderate association was found between parents' psychopathology and parenting behavior. Research has indicated that parents with mental health problems are more prone to maladaptive parenting strategies than healthy parents e.g., [59]. These associations between child psychopathology, parental psychopathology, and parenting behaviors might be interdependent: Both behavioral problems of the child and psychological problems of the parent might reinforce dysfunctional parenting behaviors, which might in turn act as a considerable family stressor for the aforementioned factors [60]. Overall, the findings of the present analysis support the concurrent validity and reliability of the FPNE. The short and comprehensive instrument can be used across genders and ages.

A strength of this study lies in the large sample combined from five projects with school-aged children with mainly externalizing problems. The FPNE was developed in a two-stage empirically-based approach, and a separate sample could be used for the exploratory and confirmatory factor analyses. A further strength is the use of different statistical methods to identify the factor structure that best fit the data (EFA; CFA, ESEM). Moreover, a preliminary validation of the FPNE with several other instruments was performed and measurement invariance for gender and age of child was also tested.

In addition to the strengths of our study, some limitations and future directions should be considered. First, as our sample consisted of school-aged children (6.00 to 12.11years), mainly with externalizing symptoms, AD symptoms, and a small group without psychological problems, the results cannot be generalized to samples with different characteristics. Future studies should therefore conduct cross-validation with nonclinical samples or samples with a wider age range and with children with other mental health conditions. Second, the cross-sectional design of our study does not permit causal interpretations and investigations of developments over time. The reliability of our results should therefore be tested in a longitudinal study. Third, as only one rater perspective (i.e., parents) was analyzed, we recommend that future studies include other informants (e.g., adolescent self-report or behavioral observations) in order to examine correlations between several rating perspectives. Fourth, further validity characteristics of the FPNE need to be investigated, in addition to the concurrent validity examined in the present study e.g., correlations with other scales of established parenting questionnaires. Additionally, assessing predictive validity would provide further insights into the instrument's effectiveness over time. Other reliability indicators such as test–retest reliability could also be considered, although the two FPNE scales showed good consistency in our study. Fifthly, in this study we have focused on a correlated two-factor model of parenting that includes a positive and negative factor. In order to gain a more comprehensive understanding of the complexity of parenting, we suggest that more intensive analyses of the underlying structures be conducted in the future. Differentiated insights into the structure of variance explanation could be gained by comparing the correlated two-factor model with other structural equation models, such as higher order CFA and ESEM or bifactor CFA and ESEM. The observed relationship between positive and negative parenting may indicate that there is a common general factor influencing parenting patterns. This suggests that parenting should be viewed as a continuum in which positive and negative aspects are closely linked. In terms of model fit, other coefficients such as omega hierarchical (ωh), explained common variance (ECV) and percent of uncorrected correlations (PUC) could provide crucial information about model quality. To date, we are not aware of any study that has investigated the underlying structure of positive and negative parenting in such depth. Initial approaches to investigating two-factor models in relation to positive aspects of parenting can be found in Martini et al. [61], whose study successfully validated a hierarchical model. Sixth, another important area that could be considered in future studies is the additional use of item response theory (IRT) analyses e.g., [62]. While the present paper has focused on confirmatory factor analysis framework (CFA) to validate the factor structure of parenting, IRT provides a complementary perspective by examining the precision of individual items in relation to different ability levels. Future research could combine these two approaches to more comprehensively assess both structural validity (CFA) and item precision (IRT), as suggested in the literature e.g., [63]. Such a hybrid approach could provide valuable insights, in particular by comparing whether the two methods produce consistent results in terms of measurement quality, and to what extent differences in the results might indicate potential for improvement in item construction.

In sum, the present article describes the development of a brief and evidence-based instrument to assess parenting within a binary framework, as suggested by the parenting literature [53]. The FPNE consists of two valid and reliable scales (Positive Parenting, Negative Parenting) that correspond to the current state of research. The analyses revealed strong psychometric properties of the instrument and an invariant factor structure across child age and gender groups. Positive and negative parenting proved to be clearly distinguishable and conceptually independent. Both dimensions are represented by specific aspects of parenting without significant overlaps. In practice, this distinction allows for targeted interventions, enabling professionals to separately address and improve positive and negative parenting practices. Consequently, the FPNE can be used in clinical and research settings as a time- and cost-efficient tool to obtain a differentiated and clear view of parenting behaviors, which in turn supports the development of precise parenting interventions.

## Supplementary Information

Additional file1 (DOCX 241 kb)

Additional file2 (DOCX 290 kb)

Additional file3 (DOCX 253 kb)

## Data Availability

The data that support the findings of this study are available from the corresponding author, VH, upon reasonable request.

## Abbreviations

AD: Affective dysregulation
ADHD: Attention-deficit/hyperactivity disorder
APQ: Alabama parenting questionnaire
CAPI: Child abuse potential inventory
CBCL 6-18R: Child behavior checklist for ages 6–18
CFA: Confirmatory factor analysis
CFI: Comparative fit index
DASS-21: Depression anxiety stress scales
DISYPS-II: Diagnostic system for mental disorders in children and adolescents according to ICD-10 and DSM-4
DISYPS-III: Diagnostic system for mental disorders in children and adolescents according to ICD-10 and DSM-5
DSM-5: Diagnostic and statistical manual 5th edition
EFA: Exploratory factor analyses
ESEM: Exploratory structural equation modeling
FBB-ADHS: The symptom checklists for attention-deficit/hyperactivity disorder
FBB-SSV: The symptom checklists for oppositional defiant disorder and conduct disorder
FPNE: Assessment scale of positive and negative parenting behavior
FZEV: Questions on parenting
HKD: Hyperkinetic disorder
ICD-10: International classification of diseases 10th edition
MAP: Velicer’s minimum average partial test
MAPS: Multidimensional assessment of parenting scale
MCBS: Management of Children’s behavior scale - revised
NP: Negative parenting
ODD: Oppositional defiant disorder
PAM: Parenting alliance measure
PAF: Principal axis factor analysis
PP: Positive parenting
PPS: Parent practices scale
PRCI: Parenting-child relationship inventory
PS: Parenting scale
RMSEA: Root mean square error of approximation
SRMR: Standardized root mean square residual
TLI: Tucker–Lewis index
WLSMV: Weighted least squares with mean and variance adjustment
