# Supplementary material for: Psychometric properties of the parent-rated assessment scale of positive and negative parenting behavior (FPNE) in a German sample of school-aged children
Source: Child Adolesc Psychiatry Ment Health. 2024 Dec 16;18:157. doi: 10.1186/s13034-024-00850-9 (PMC11648292; doi:10.1186/s13034-024-00850-9)
Supplement: Supplementary file 2 — Additional file2 (DOCX 290 kb) [file 13034_2024_850_MOESM2_ESM.docx]

**Supplementary material**

**Table S1**

*Overview of Existing Parenting Assessments that are Part of MAPS or Showed Acceptable Psychometric Properties Evaluated According to Hurley et al.’s (8) Review*

| **Authors (Year)** | **Parenting Measures** | **Scale** | **No. of Items** | **Part of MAPS** | **Hurley et al., 2014** | **Subscales** |
| --- | --- | --- | --- | --- | --- | --- |
| Parent & Forehand, 2017 (9) | The Multidimensional Assessment of Parenting Scale (MAPS) | 5-point | 34 |  |  | Broadband Positive Parenting  Proactive Parenting  Positive Reinforcement  Warmth  Supportiveness  Broadband Negative Parenting  Hostility  Lax Control  Physical Control |
| Shelton, Frick, & Wotton, 1996 (10) | Alabama Parenting Questionnaire (APQ) | 5-point | 42 | ✓ | ✓ | Positive Involvement  Positive Parenting  Monitoring  Inconsistent Discipline  Corporal Punishment |
| Robinson et al., 1995 (11) | Parenting Style Questionnaire (PRQ) | 5-point | 62 | ✓ |  | Authoritative  Authoritarian  Permissive |
| Arnold et al., 1993 (12) | Parenting Scale (PS) | 7-point | 30 | ✓ | ✓ | Laxness  Overreactivity  Hostility |
| Perepletchikova & Kazdin, 2004 (13) | Management of Children’s Behavior Scale (MCBS) | 3-point | 38 | ✓ |  | Inept Parenting |
| Schaefer, 1965 (14) | Children’s Report of Parenting Behavior Inventory (CRPBI) | 3-point | 30 | ✓ |  | Acceptance vs. Rejection  Psychological Control vs. Autonomy  Firm Control vs. Autonomy |
| Lovejoy et al., 1999 (15) | Parent Behavior Inventory (PBI) | 6-point | 20 | ✓ |  | Supportive/Engaged  Hostile/Coercive |
| McEachern et al., 2012 (16) | Parenting Young Children (PARYC) | 7-point | 21 | ✓ |  | Setting Limits  Supporting Positive Behavior  Proactive Parenting |
| Stattin & Kerr, 2000 (17) | Parental Monitoring (PM) | 5-point | 9 | ✓ |  | Monitoring |
| Gerard, 1994 (18) | Parent–Child Relationship Inventory (PRCI) | 4-point | 78 |  | ✓ | Parental Support  Satisfaction with Parenting Involvement  Communication  Limit Setting  Autonomy  Role Orientation |
| Milner’s, 1986, 1994, 2004 (19-21) | Child Abuse Potential Inventory (CAPI) | 2-point | 160 |  | ✓ | Distress  Rigid Expectations  Unhappiness  Problems relating with one’s Child, Family, and Others |
| Abidin & Konold, 1999; Konold & Abidin, 2001 (22, 23) | Parenting Alliance Measure (PAM) | 5-point | 20 |  | ✓ | Parenting Alliance |

**Table S2**

*Inclusion and Exclusion Criteria of the Five Participating Projects*

|  | **ADOPT** (1) | **Self-help comparison study** (2) | **Enhancement** (3) | **ESCAschool** (4) | **WASH** (5) |
| --- | --- | --- | --- | --- | --- |
| **Inclusion criteria** |  |  |  |  |  |
| Child age | 8;0 to 12;11 years | 4;0 to 11;11 years | 6;0 to 12;11 years | 6;0 to 11;11 years | 6;0 to 12;11 years |
| Kindergarten/school | all school types | kindergarten, primary school, or special school | school | school |  |
| Child diagnosis/symptoms | AD symptoms (within the AD group) (DADYS-PI) based on parent interview | ADHD or ODD symptoms or diagnosis according to DSM-IV-TR | ADHD according to  DSM-IV | ADHD according to  DSM-5 | ADHD or ODD symptoms or diagnosis according to DSM-5 |
| Other |  |  | - stable dose of methylphenidate for at least the previous 2 months and no change of medication or dose planned  - child functional impairment in at least one of the domains captured by WFIRS-P | - informed consent of both parents or guardians and assent of the child |  |
| Parent aspects | - willingness and ability to participate  - sufficient German-language skills  - ADOPT-ONLINE: Availability of internet access | - sufficient motivation  - willingness  - sufficient German-language skills | - sufficient motivation  - sufficient German-language skills  - not already involved in a possible psychotherapy for their child | - sufficient German-language skills | - sufficient German-language skills |
| **Exclusion criteria** |  |  |  |  |  |
| Child IQ | - child’s intelligence below average |  |  | - child intelligence below average (IQ < 80) |  |
| Child Diagnosis/symptoms | - mental disorder other than comorbid conditions is the primary disorder and the main cause of AD (e.g., autism spectrum disorder) | - indication or previous diagnosis of borderline intellectual functioning, intellectual disability, pervasive developmental disorder |  | - child pervasive developmental disorder  - schizophrenia  - bipolar disorder  - severe depressive episode  - epilepsy or heart disease | - mental retardation  - autism spectrum disorder |
| Treatment | - current or planned intensive behavioral therapy (ADOPT Online)/ psychotherapy (ADOPT Treatment and ADOPT Institution) or behavioral PMT on a weekly/ biweekly basis | - inpatient treatment or another intensive treatment |  | - current or planned intensive behavior therapy for child ADHD or ODD on a weekly basis | - indication for inpatient treatment |
| Medication |  | - planned start or discontinuation of psychopharmacological treatment or a change in dosage during the intervention period of the trial (first 5 months after baseline) |  | - known non-response of the child to all standard ADHD medication  - psychotropic medication of the child other than for the treatment of ADHD, or neuroleptic medication other than for the treatment of disturbances of impulse control |  |

*Note.* WFIRS-P = German adaptation of the Weiss Functional Impairment Rating Scale–Parent Report (6), DADYS-PI = Diagnostic Tool for Affective Dysregulation in Children – Parent Interview (7).

**Table S3**

*Overview of all 41 Items, the First Selection Phase by Imort and Colleagues (28) (38 Items Result) and the Second Selection Phase of this Study (23 Items Result)*

| *N* = 149 (Imort et al., 2014) | *N* = 1879 | FPNE item | Original item number | Original item / translation of new item | Origin |
| --- | --- | --- | --- | --- | --- |
| 01 | 01 | I show my child appreciation when he does things I like. | 05 | I show my child appreciation when he does things I like. | FZEV |
| 02 | 02 | I talk to my child. | 09 | I talk to my child. | FZEV |
| 03 | -- | If my child does an unexpected task or chore, I will make a big fuss about it. | 21 | If my child does an unexpected task or chore, I will make a big fuss about it. | MCBS |
| 04 | 03 | Ich habe Spaß mit meinem Kind. | 08 | I have fun with my child. | FZEV |
| 05 | 04 | I punish my child for doing something one day, but ignore it the next day. | 01 | I punish my child for doing something one day, but ignore it the next day. | MCBS |
| 06 | 05 | Ich bin häufig genervt, wenn mein Kind mit mir spielen möchte. |  | I’m often irritated if my child wants to play with me. | new |
| 07 | 06 | Jedes Mal, wenn ich mein Kind zu bestimmten Dingen auffordere, diskutiere ich lange mit ihm darüber. |  | Whenever I ask my child to do certain things, I discuss it with him/her for a long time. | new |
| 08 | 07 | Ob ich eine Strafe verhänge oder nicht, das hängt häufig von meiner Stimmung ab. |  | Whether or not I impose a punishment often depends on my mood. | new |
| 09 | 08 | I believe that if my child had misbehaved during the day, none of his good behavior should be rewarded. | 31 | I believe that if my child had misbehaved during the day, none of his good behavior should be rewarded. | MCBS |
| 10 | -- | Ich versuche, meinem Kind kurze und knappe Anweisungen zu geben, die es dann leichter umsetzen kann. – umkodiert |  | I try to give my child short and succinct instructions that he/she can easily implement. – reverse-recoded | new |
| 11 | -- | Bei uns zu Hause gibt es ein paar Regeln, an die sich jeder (auch die Erwachsenen) zu halten hat. – umkodiert |  | We have a few rules at home, which everyone (including the adults) has to stick to. – reverse-recoded | new |
| 12 | -- | Ich bin mir mit meinem Partner (oder einer anderen wichtigen Person) häufig nicht einig, ob ein bestimmtes Verhalten meines Kindes bestraft werden soll, oder nicht. |  | I often disagree with my partner (or another important person) about whether or not a particular behavior of my child should be punished. | new |
| 13 | -- | Ich vergesse häufig, mein Kind zu loben, wenn es etwas gut gemacht hat. - umkodiert |  | I often forget to praise my child when he/she has done something good. – reverse-coded | new |
| 14 | -- | When I review my child’s report card, I tell him how proud I am of his work. | 16 | When I review my child’s report card, I tell him how proud I am of his work. | MCBS |
| 15 | -- | Ich setze mich ab und zu mit meiner Familie zusammen, um die Regeln, die in unserer Familie gelten sollen, zu besprechen. - umkodiert |  | From time to time, I sit down with my family to discuss the rules that should apply in our family. -reverse-recoded | new |
| 16 | -- | If my child brings home a test from school and he has made a small improvement, I will tell him how proud I am of his grade. | 24 | If my child brings home a test from school and he has made a small improvement, I will tell him how proud I am of his grade. | MCBS |
| 17 | 09 | Ich sage meinem Kind etwas Nettes. | 10 | I say something nice to my child. | FZEV |
| 18 | 10 | I threaten to punish my child for his misbehavior, but I do not follow through. | 02 | I threaten to punish my child for his misbehavior, but I do not follow through. | MCBS |
| 19 | 11 | If my child misbehaves, I will swear at him or call him names. | 10 | If my child misbehaves, I will swear at him or call him names. | MCBS |
| 20 | -- | Wenn mein Kind eine Strafe erhalten hat, fällt mir danach ein freundlicher Umgang mit ihm schwer. - umkodiert |  | When my child has received a punishment, I find it hard to be friendly to him/her afterwards. – reverse-coded | new |
| 21 | -- | I take away a privilege for a week or more when my child misbehaves. | 11 | I take away a privilege for a week or more when my child misbehaves. | MCBS |
| 22 | 12 | Ich bemerke bei mir selbst, dass ich in Stress- oder Konfliktsituationen auf mein Kind einrede. |  | I notice in myself that I talk insistently at my child in stressful or conflict situations. | new |
| 23 | -- | Ich lobe mein Kind nicht so häufig, weil ich finde, dass vieles selbstverständlich sein sollte. - umkodiert |  | I don’t praise my child very often because I think lots of things should go without saying. – reverse-coded | new |
| 24 | 13 | Ich spiele mit meinem Kind. | 01 | I play with my child. | FZEV |
| 25 | 14 | Ich schmuse mit meinem Kind. | 11 | I cuddle with my child. | FZEV |
| 26 | 15 | Ich unternehme etwas mit meinem Kind. | 07 | I do things with my child. | FZEV |
| 27 | 16 | I take away a privilege but if my child whines or complains, I will give it back. | 03 | I take away a privilege but if my child whines or complains, I will give it back. | MCBS |
| 28 | -- | Ich schaffe es gut, in Stresssituationen eher wenig und ruhig mit meinem Kind zu sprechen. |  | I manage well to speak rather little and quietly with my child in stressful situations | new |
| 29 | 17 | Ich lache gemeinsam mit meinem Kind. | 02 | I laugh with my child. | FZEV |
| 30 | -- | I ground my child for days at a time when my child disobeys. | 12 | I ground my child for days at a time when my child disobeys. | MCBS |
| 31 | 18 | Ich spiele mit meinem Kind Rollen- oder Puppenspiele. | 12 | I do role-plays or puppet shows with my child. | FZEV |
| 32 | 19 | Ich lobe mein Kind. | 04  14 | I praise my child.  I often praise my child. | FZEV MCBS |
| 33 | 20 | Ich erzähle meinem Kind etwas von mir. | 03 | I tell my child things about myself. | FZEV |
| 34 | 21 | Wenn mein Kind mir etwas zeigen will, nehme ich mir Zeit dafür. | 13 | If my child wants to show me something, I take the time for it. | FZEV |
| 35 | -- | When spanking my child, I have used other things besides my hand. | 07 | When spanking my child, I have used other things besides my hand. | MCBS |
| 36 | 22 | Wenn mein Kind etwas gemacht hat, wozu ich es aufgefordert habe, sage ich manchmal: „Warum nicht immer so?“. |  | If my child does something I’ve asked, I sometimes say: “Why don’t you always do that”? | new |
| 37 | 23 | Wenn mein Kind zu mir kommt und ich beschäftigt bin, versuche ich es in meine Tätigkeit einzubeziehen. | 06 | If my child comes to me and I’m busy, I try to include him/her in my activity. | FZEV |
| 38 | -- | If my child does his chores, I will recognize his behavior in some manner. | 20 | If my child does his chores, I will recognize his behavior in some manner. | MCBS |
| -- |  | Ich achte darauf, meinem Kind nicht aus der Ferne Aufforderungen zuzurufen, da es diese häufig schlechter behalten kann. |  | I make sure I don’t shout requests to my child from a distance, because he/she often finds this harder to remember. | new |
| -- |  | Ich halte es für unnötig, meinem Kind bestimmte Regeln mehrmals zu erklären. |  | I find it unnecessary to explain certain rules to my child several times. | new |
| -- |  | Ich achte darauf, mein Kind nicht zu häufig zu belohnen, damit ich es nicht verwöhne. |  | I make sure I don’t praise my child too often so I don’t spoil him/her. | new |

*Note.* -- = excluded items in analysis of Imort et al. (28) and this study**,** FZEV = Fragen zum Erziehungsverhalten (25, 26), MCBS = Management of Children’s Behavior Scale – Revised (13, 27), new = self-constructed item.

**Table S4**

*Participant Demographic Characteristics for Total Sample and Subsamples*

|  | Total | ADOPT | Self-help comparison (SHC) | Enhancement | ESCAschool | WASH | test statistics | *effect size* | *pairwise* | *effect size* |
| --- | --- | --- | --- | --- | --- | --- | --- | --- | --- | --- |
| *N* | 1879 | 695 | 104 | 114 | 559 | 407 |  |  |  |  |
| child variables |  |  |  |  |  |  |  |  |  |  |
| age (years):  *M* (*SD*) [range] | 9.70 (1.64) [7.48] | 10.62 (1.36) [5.29] | 8.79 (1.31) [5.71] | 9.80 (1.58) [7.12] | 8.88 (1.44) [5.84] | 9.47 (1.61) [6.99] | *H*(4)=393.15; *p*<.05 | *d*=1.02 | SHC-WASH  SHC-Enhancement SHC-ADOPT  ESCA-WASH  ESCA-Enhancement ESCA-ADOPT  WASH-ADOPT Enhancement-ADOPT | *r*=.17 *r*=.31 *r*=.38 *r*=.18  *r*=.21 *r*=.52 *r*=.33 *r*=.17 |
| gender (male) | 73.4% | 60.7% | 79.8% | 80.7% | 80.7% | 81.6% | *H*(4)=91.68; *p*<.05 | *V*=0.22 |  |  |
| enrolled in school |  |  |  |  |  |  | *H*(3)=124.81; *p*<.05 | *d*=0.54 | ESCA-WASH ESCA-ADOPT Enhancement-ADOPT WASH-ADOPT | *r*=.13 *r*=.31 *r*=.16 *r*=.18 |
| type of school |  |  |  |  |  |  |  |  |  |  |
| primary school | 61.7% | 42.2% | -- | 65.8% | 73.5% | 65.4% |  |  |  |  |
| special school | 7.3% | 10.1% | -- | 3.5% | 6.6% | 4.9% |  |  |  |  |
| secondary school | 11.3% | 13.1% | -- | 15.8% | 7.5% | 8.6% |  |  |  |  |
| high school | 19.5% | 32.2% | -- | 16.0% | 9.6% | 20.1% |  |  |  |  |
| other | 1.6% | 2.4% | -- | 0.9% | 2.0% | 1.0% |  |  |  |  |
| special educational needs (yes) | 13.5% | 7.9% | 10.6% | 25.4% | 12.9% | 21.1% | *H*(4)=43.71; *p*<.001 | *V*=0.16 |  |  |
| diagnosis ADHD/ODD incl. HKD (yes) | 77.6% | 43.5% | 66.3% | 100% | 100% | 77.6% | *H*(4)=642.70; *p*<.001 | *V*=0.59 |  |  |
| ADHD medication (yes) | 32.4% | 10.8% | -- | -- | 32.6% | 53.8% | *H*(4)=1414.11; *p*<.001 | *V*=0.89 |  |  |
| parent variables |  |  |  |  |  |  |  |  |  |  |
| age participant (years): M *(SD)* [range] | 41.40 (6.32) [42.77] | 43.75 (6.04) [42.65] | 38.40 (6.22) [28.00] | 40.00 (6.24) [30.08] | 39.80 (6.18) [33.30] | 41.28 (5.83) [36.00] | *H*(4)=138.95; *p*<.001 | *d*=0.58 | SHC-WASH SHC-ADOPT ESCA-WASH ESCA-ADOPT Enhancement-ADOPT WASH-ADOPT | *r*=.17 *r*=.27 *r*=.11 *r*=.29 *r*=.19 *r*=.18 |
| single-parent status (yes) | 17.2% | 11.9% | 23.1% | -- | 15.0% | 18.7% | *H*(3)=317.71; *p*<.001 |  |  |  |
| country of origin (Germany) | 87.9% | 76.7% | 95.2% | -- | 86.7% | 92.9% | *H*(3)=13.29; *p*=.004 | *V*=0.09 |  |  |
| language spoken at home (German) | 96.0% | 97.4% | 97.1% | 98.2% | 91.9% | 98.0% | *H*(4)=23.02; *p*<.001 | *V*=0.11 |  |  |

*Note*. *M* = mean, *SD* = standard deviation, ADHD = Attention Deficit Hyperactivity Disorder, ODD = Oppositional Defiant Disorder, HKD = Hyperkinetic Disorder, Special educational needs defined as “a learning difficulty which calls for special educational provision to be made” (24, p.6), test statistics for five groups are based on Kruskal-Wallis Test, *H* = Kruskal-Wallis H, *d* = Cohen’s *d*, *V* = Cramer’s *V*.

**Figure F1**

*Velicer’s Minimum Average Partial (MAP) test results for 38 items version*

*Note*. Blue line: the average squared partial correlations; Red line: the average fourth power partial correlations.

**Figure F2**

*Velicer’s Minimum Average Partial (MAP) test results for 23 items version*

*Note*. Blue line: the average squared partial correlations; Red line: the average fourth power partial correlations.

**Figure F3**

*Parallel analysis results for 38 items version*

**

*Note.* Blue line: Empirical eigenvalues; Red line: Random eigenvalues.

**Figure F4**

*Parallel analysis results for 23 items version*

**

*Note.* Blue line: Empirical eigenvalues; Red line: Random eigenvalues.

**Figure F5**

*Stepwise Item Selection Process*

#

Total item pool = 41 items (The item "I praise my child." overlaps in the FZEV and MCBS scale)

Elimination of 15 items based on exploratory factor analysis

FPNE

Total item pool = 23 items

Combined items of FZEV (13 items) and MCBS/self-constructed (10 items)

Preliminary FPNE

Total item pool = 38 items

Elimination of 3 items based on preliminary analysis (Imort et al., 2014)

Total FZEV scale, 13 items

16 items constructed by a focus group of experts in behavioral child and adolescent psychotherapy

13 items of the MCBS scale

*Note*. FZEV = Questions on Parenting (German: Fragen zum Erziehungsverhalten; 25, 26), MCBS = Management of Children’s Behavior Scale – Revised (13, 27), FPNE = Assessment of Positive and Negative Parenting Behavior.

**Table S5**

*Exploratory factor analysis results with specification of 2 factors with 38 items by split-half sample (N=917)*

|  | Total | |
| --- | --- | --- |
|  | PP | NP |
| 32. I praise my child. | **.77** | .04 |
| 17. I say something nice to my child. | **.76** | .06 |
| 29. I laugh with my child. | **.66** | -.06 |
| 26. I do things with my child. | **.63** | .03 |
| 25. I cuddle with my child. | **.62** | .25 |
| 14. When I review my child’s report card, I tell him how proud I am of his work. | **.62** | .08 |
| 16. If my child brings home a test from school and he has made a small improvement, I will tell him how proud I am of his grade. | **.62** | .14 |
| 1. I show my child appreciation when he does things I like. | **.60** | -.09 |
| 4. I have fun with my child. | **.60** | -.14 |
| 33. I tell my child things about myself. | **.60** | .07 |
| 2. I talk to my child. | **.59** | -.03 |
| 38. If my child does his chores, I will recognize his behavior in some manner. | **.57** | -.03 |
| 34. If my child wants to show me something, I take the time for it. | **.53** | -.16 |
| 3. If my child does an unexpected task or chore, I will make a big fuss about it. | **.52** | .13 |
| 13. I often forget to praise my child when he/she has done something good. – reverse-coded | **.51** | -.23 |
| 23. I don’t praise my child very often because I think lots of things should go without saying. – reverse-coded | **.45** | -.18 |
| 37. If my child comes to me and I’m busy, I try to include him/her in my activity. | **.37** | -.10 |
| 31. I do role-plays or puppet shows with my child. | **.31** | -.03 |
| 15. From time to time, I sit down with my family to discuss the rules that should apply in our family. -reverse-recoded |  | .13 |
| 10. I try to give my child short and succinct instructions that he/she can easily implement. – reverse-recoded |  | .01 |
| 18. I threaten to punish my child for his misbehavior, but I do not follow through. | .05 | **.72** |
| 8. Whether or not I impose a punishment often depends on my mood. | -.03 | **.67** |
| 22. I notice in myself that I talk insistently at my child in stressful or conflict situations. | -.03 | **.62** |
| 5. I punish my child for doing something one day, but ignore it the next day. | .11 | **.59** |
| 7. Whenever I ask my child to do certain things, I discuss it with him/her for a long time. | -.03 | **.57** |
| 19. If my child misbehaves, I will swear at him or call him names. | -.07 | **.55** |
| 27. I take away a privilege but if my child whines or complains, I will give it back. | .08 | **.54** |
| 12. I often disagree with my partner (or another important person) about whether or not a particular behavior of my child should be punished. | .03 | **.49** |
| 28. I manage well to speak rather little and quietly with my child in stressful situations | .20 | **.42** |
| 6. I’m often irritated if my child wants to play with me. | -.16 | **.41** |
| 20. When my child has received a punishment, I find it hard to be friendly to him/her afterwards. – reverse-coded | .30 | **-.39** |
| 36. ‘If my child does something I’ve asked, I sometimes say: ‘Why don’t you always do that’? | -.02 | **.37** |
| 9. I believe that if my child had misbehaved during the day, none of his good behavior should be rewarded. | -.22 | .28 |
| 21. I take away a privilege for a week or more when my child misbehaves. | .04 | .26 |
| 35. When spanking my child, I have used other things besides my hand. | -.09 |  |
| 11. We have a few rules at home, which everyone (including the adults) has to stick to. – reverse-recoded | -.18 |  |
| 30. I ground my child for days at a time when my child disobeys. | .03 |  |

*Note.* Principal axis factor analysis with oblimin rotation. PP = Positive Parenting. NP = Negative Parenting. *R^2^* = 28.03.

**Table S6**

*Final exploratory factor analysis results with specification of 2 factors (N=917)*

|  | Factorial loads based on  Pearson correlation matrix | | Factorial loads based on  polychoric correlation matrix | |
| --- | --- | --- | --- | --- |
|  | PP | NP | PP | NP |
| 17. I say something nice to my child. | **.70** |  | **.78** |  |
| 29. I laugh with my child. | **.69** |  | **.77** |  |
| 4. I have fun with my child. | **.68** |  | **.76** |  |
| 32. I praise my child. | **.66** |  | **.76** |  |
| 25. I cuddle with my child. | **.61** | -.22 | **.66** | -.24 |
| 26. I do things with my child. | **.61** |  | **.67** |  |
| 2. I talk to my child. | **.60** |  | **.72** |  |
| 33. I tell my child things about myself. | **.60** |  | **.66** |  |
| 24. I play with my child. | **.54** |  | **.63** |  |
| 1. I show my child appreciation when he does things I like. | **.52** | -.14 | **.62** | -.16 |
| 34. If my child wants to show me something, I take the time for it. | **.49** | -.17 | **.58** | -.19 |
| 37. If my child comes to me and I’m busy, I try to include him/her in my activity. | **.33** | -.10 | **.38** | -.11 |
| 31. I do role-plays or puppet shows with my child. | **.33** |  | **.39** |  |
| 18. I threaten to punish my child for his misbehavior, but I do not follow through. |  | **.72** |  | **.77** |
| 8. Whether or not I impose a punishment often depends on my mood. |  | **.70** |  | **.75** |
| 5. I punish my child for doing something one day, but ignore it the next day. |  | **.63** |  | **.71** |
| 27. I take away a privilege but if my child whines or complains, I will give it back. |  | **.56** |  | **.63** |
| 22. I notice in myself that I talk insistently at my child in stressful or conflict situations. | -.10 | **.51** | -.11 | **.54** |
| 7. Whenever I ask my child to do certain things, I discuss it with him/her for a long time. |  | **.51** |  | **.54** |
| 19. If my child misbehaves, I will swear at him or call him names. | -.13 | **.45** | -.14 | **.48** |
| 6. I’m often irritated if my child wants to play with me. | -.19 | **.40** | -.22 | **.45** |
| 36. ‘If my child does something I’ve asked, I sometimes say: ‘Why don’t you always do that’? |  | **.35** |  | **.38** |
| 9. I believe that if my child had misbehaved during the day, none of his good behavior should be rewarded. | -.17 | **.26** | -.21 | **.33** |

*Note.* Principal axis factor analysis with oblimin rotation. PP = Positive Parenting. NP = Negative Parenting. *R^2^* = 32.34 based on Pearson correlation matrix. *R^2^* = 41.05 based on polychoric correlation matrix.

**Table S7**

*Item statistics, item-level correlations, scale statistics and test statistics of the final FPNE version (N=1879)*

| **Item** | ***M*** | ***SD*** | **frequency** | | | | ***r_itc_* to PP** | ***r_itc_* to NP** | **test statistics** | **effect size** | **pairwise/contrasts** | **effect size** |
| --- | --- | --- | --- | --- | --- | --- | --- | --- | --- | --- | --- | --- |
|  |  |  | **1** | **2** | **3** | **4** |  |  |  |  |  |  |
| 1. show recognition | 3.46 | 0.58 | 0.1 | 4.3 | 44.9 | 50.7 | .51 |  | *H*(4)=63.91; *p*<.001 | *d*=.36 | ADOPT-SHC ADOPT-Enhancement ADOPT-ESCA  ADOPT-WASH | *r* =.18 *r* =.17 *r* =.15 *r* =.16 |
| 2. communicate with the child | 3.52 | 0.55 | 0 | 2.9 | 42.5 | 54.6 | .54 |  | *H*(4)=68.33; *p*<.001 | *d*=.37 | ADOPT-SHC ADOPT-Enhancement ADOPT-ESCA  ADOPT-WASH  ESCA-WASH | *r* =.13 *r* =.09 *r* =.23 *r* =.10 *r* =.12 |
| 3. have fun with the child | 2.99 | 0.71 | 0.5 | 23.9 | 51.4 | 24.2 | .64 |  | *H*(4)=84.44; *p*<.001 | *d*=.42 | ADOPT-SHC ADOPT-Enhancement ADOPT-ESCA  ADOPT-WASH | *r* =.22 *r* =.18 *r* =.19 *r* =.18 |
| 9. say something nice | 3.26 | 0.65 | 0,1 | 11.1 | 51.1 | 37.7 | .63 |  | *H*(4)=23.66; *p*<.001 | *d*=.21 | SHC-ADOPT  SHC-WASH ESCA-ADOPT | *r* =.12 *r* =.15 *r* =.09 |
| 13. play with the child | 2.61 | 0.65 | 0.3 | 47.5 | 43.2 | 9.0 | 52 |  | *H*(4)=67.85; *p*<.001 | *d*=.38 | ESCA-WASH ESCA-ADOPT SHC-ADOPT | *r* =.17  *r* =.22 *r* =.11 |
| 14. cuddle with the child | 3.16 | 0.78 | 2.3 | 16.6 | 43.5 | 37.5 | .51 |  | *H*(4)=5.92.; *p*=.205 | *d*=.06 |  |  |
| 15. do things with the child | 2.98 | 0.67 | 0.3 | 22.9 | 55.5 | 21,4 | .54 |  | *H*(4)=17.92; *p*=.001 | *d*=.17 | ESCA-ADOPT | *r* =.11 |
| 17. laugh with the child | 3.11 | 0.68 | 0.2 | 17.3 | 53.6 | 29.0 | .65 |  | *H*(4)=74.94; *p*<.001 | *d*=.40 | SHC-WASH ESCA-WASH ADOPT-SHC ADOPT-Enhancement ADOPT-ESCA  ADOPT-WASH | *r* =.14 *r* =.11 *r* =.15  *r* =.13 *r* =.22 *r* =.11 |
| 18. do role-play/ puppet shows | 1.51 | 0.65 | 56.4 | 37.4 | 5.1 | 1.1 | .33 |  | *H*(4)=5.45; *p*=.244 |  |  |  |
| 19. praise the child | 3.16 | 0.64 | 0.1 | 13.4 | 57.2 | 29.4 | .63 |  | *H*(4)=32.63; *p*<.001 | *d*=.25 | SHC-ADOPT ESCA-WASH ESCA-ADOPT | *r* =.12 *r* =.11  *r* =.14 |
| 20. talk about themselves | 2.90 | 0.68 | 0.9 | 25.5 | 55.7 | 17.8 | .52 |  | *H*(4)=23.22; *p*<.001 | *d*=.20 | SHC-ADOPT ESCA-ADOPT WASH-ADOPT | *r* =.12 *r* =.11 *r* =.10 |
| 21. take time occasionally | 3.09 | 0.56 | 0.1 | 11.3 | 68.0 | 20.6 | .51 |  | *H*(4)=26.84; *p*<.001 | *d*=.22 | Enhancement-ADOPT ESCA-ADOPT | *r* =.10 *r* =.13 |
| 23. involve the child in activities | 2.40 | 0.65 | 3.8 | 57.8 | 32.9 | 5.4 | .35 |  | *H*(4)=26.91; *p*<.001 | *d*=.22 | SHC-ADOPT SHC-ESCA WASH-ESCA | *r* =.11 *r* =.16 *r* =.12 |
| 4. inconsistent discipline | 2.00 | 0.65 | 18.8 | 64.6 | 14.3 | 2.2 |  | .50 | *H*(4)=40.57; *p*<.001 | *d*=.28 | ADOPT-SHC ADOPT-Enhancement ADOPT-ESCA  ADOPT-WASH | *r* =.12 *r* =.10 *r* =.13 *r* =.16 |
| 5. frequently annoyed | 1.89 | 0.58 | 22.5 | 66,9 | 9.8 | 0.7 |  | .39 | *H*(4)=34.36; *p*<.001 | *d*=.26 | ADOPT-SHC ADOPT-Enhancement ADOPT-ESCA  ADOPT-WASH | *r* =.14 *r* =.12 *r* =.10 *r* =.13 |
| 6. long discussions in the case of requests | 2.57 | 0.88 | 10.5 | 38.3 | 35.3 | 15.9 |  | .46 | *H*(4)=80.14; *p*<.001 | *d*=.41 | ADOPT-SHC ADOPT-Enhancement ADOPT-ESCA  ADOPT-WASH | *r* =.16 *r* =.12 *r* =.17 *r* =.24 |
| 7. mood-dependent punishment | 1.91 | 0.70 | 28.3 | 53.7 | 16.7 | 1.3 |  | .56 | *H*(4)=29.75; *p*<.001 | *d*=.24 | ADOPT-ESCA ADOPT-WASH | *r* =.11 *r* =.14 |
| 8. no reward following misbehavior during the day | 1.39 | 0.57 | 64.9 | 31.6 | 3.1 | 0.5 |  | .31 | *H*(4)=15.48; *p*=.004 | *d*=.16 | ADOPT-WASH ADOPT-Enhancement | *r* =.09 *r* =.11 |
| 10. empty threats | 1.93 | 0.72 | 27.4 | 55.3 | 14.7 | 2.6 |  | .58 | *H*(4)=59.36; *p*<.001 | *d*=.35 | ADOPT-SHC ADOPT-Enhancement ADOPT-ESCA  ADOPT-WASH | *r* =.16 *r* =.15 *r* =.13 *r* =.19 |
| 11. verbal hostility | 2.07 | 0.82 | 26.9 | 43.3 | 26.1 | 3.7 |  | .40 | *H*(4)=78.49; *p*<.001 | *d*=.35 | ADOPT-SHC ADOPT-Enhancement ADOPT-ESCA  ADOPT-WASH ESCA-Enhancement | *r* =.37 *r* =.22 *r* =.13 *r* =.19 *r* =.13 |
| 12. talk insistently at the child in situations of stress/conflict | 2.34 | 0.80 | 13.6 | 45.3 | 34.3 | 6.8 |  | .48 | *H*(4)=92.87; *p*<.001 | *d*=.45 | ADOPT-SHC ADOPT-Enhancement ADOPT-ESCA  ADOPT-WASH | *r* =.23 *r* =.20 *r* =.16 *r* =.21 |
| 16. restore privileges if child whines | 1.53 | 0.65 | 54.1 | 39.2 | 5.8 | 0.9 |  | .45 | *H*(4)=22.04; *p*<.001 | *d*=.20 | ADOPT-ESCA ADOPT-SHC | *r* =.11 *r* =.11 |
| 22. ‘Why not always like this?’ | 1.82 | 0.74 | 36.7 | 46.9 | 14.5 | 1.9 |  | .32 | *H*(4)=23.07; *p*<.001 | *d*=.20 | ADOPT-ESCA | *r* =.13 |
| PP | 2.94 | 0.40 |  |  |  |  |  |  | F(4)=2.28; p<.001 | η² = .03 | ADOPT-SHC ADOPT-Enhancement ADOPT-ESCA  ADOPT-WASH SHC-WASH | *d*=1.06 *d*=.81 *d*=.72 *d*=.42 *d*=.64 |
| NP | 1.95 | 0.41 |  |  |  |  |  |  | F(4)=5.18; p<.001 | η² = .07 | ADOPT-SHC ADOPT-Enhancement ADOPT-ESCA  ADOPT-WASH | *d*=1.34 *d*=1.26 *d*=.86 *d*=1.11 |

*Note. M* = mean, *SD* = standard deviation, *r_itc_* = selectivity, PP = Positive Parenting, NP = Negative Parenting, test statistics for five groups are based on Kruskal-Wallis tests for categorical data and one-way ANOVAs for continuous data, Bonferroni post-hoc test, η² = partial eta-squared, *d* = Cohen’s *d*, *r* = Pearson *r* correlation, *H* = Kruskal-Wallis *H*.

**Table S8**

*Internal Consistencies (Cronbach’s Alpha and omega in bold) of the two FPNE Scales for the Total Sample and the five Subsamples*

|  | Total | ADOPT | Self-help comparison study | Enhance-ment | ESCAschool | WASH |
| --- | --- | --- | --- | --- | --- | --- |
| PP |  |  |  |  |  |  |
| *M* | 2.94 | 3.02 | 2.81 | 2.86 | 2.88 | 2.93 |
| *SD* | 0.40 | 0.40 | 0.39 | 0.40 | 0.38 | 0.39 |
| range | 2.23 | 2.08 | 2.00 | 1.77 | 2.23 | 2.00 |
| **ω** | **.87** | **.89** | **.89** | **.90** | **.85** | **.87** |
| α | **.86** | **.87** | **.86** | **.88** | **.85** | **.86** |
| NP |  |  |  |  |  |  |
| *M* | 1.95 | 1.81 | 2.09 | 2.07 | 1.99 | 2.04 |
| *SD* | 0.41 | 0.42 | 0.39 | 0.38 | 0.40 | 0.38 |
| range | 2.60 | 2.50 | 2.40 | 1.90 | 2.10 | 2.20 |
| **ω** | **.78** | **.82** | **.80** | **.81** | **.74** | **.74** |
| α | **.78** | **.80** | **.75** | **.72** | **.74** | **.73** |

Note. PP = Positive Parenting (13 items), NP = Negative Parenting (10 items), *N*_total_ = 1879, *n*_ADOPT_ = 695,

*n*_Self-help comparison_ = 104, *n*_Enhancement_ = 114, *n*_ESCA_ = 559, *n*_WASH_ = 407.

**Table S9**

*Item-Scale Statistics for the Positive Parenting Scale with 17 Items*

|  | Scale Mean if Item Deleted | Corrected Item-Total Correlation | Cronbach’s Alpha if Item Deleted |
| --- | --- | --- | --- |
| 32. I praise my child. | 48.64 | .69 | .879 |
| 17. I say something nice to my child. | 48.57 | .68 | .879 |
| 29. I laugh with my child. | 48.72 | .65 | .88 |
| 26. I do things with my child. | 48.87 | .60 | .881 |
| 25. I cuddle with my child. | 48.74 | .51 | .886 |
| 14. When I review my child’s report card, I tell him how proud I am of his work. | 48.40 | .55 | .883 |
| 4. I have fun with my child. | 48.88 | .62 | .881 |
| 16. If my child brings home a test from school and he has made a small improvement, I will tell him how proud I am of his grade. | 48.88 | .50 | .885 |
| 33. I tell my child things about myself. | 48.95 | .54 | .884 |
| 1. I show my child appreciation when he does things I like. | 48.39 | .58 | .883 |
| 2. I talk to my child. | 48.32 | .57 | .883 |
| 38. If my child does his chores, I will recognize his behavior in some manner. | 48.53 | .54 | .884 |
| 34. If my child wants to show me something, I take the time for it. | 48.72 | .55 | .883 |
| 24. I play with my child. | 49.20 | .53 | .884 |
| 3. If my child does an unexpected task or chore, I will make a big fuss about it. | 48.56 | .42 | .888 |
| 37. If my child comes to me and I’m busy, I try to include him/her in my activity. | 49.51 | .34 | .891 |
| 31. I do role-plays or puppet shows with my child. | 50.36 | .32 | .891 |

**Table S10**

*Intercorrelations of the two FPNE Scales for the Total Sample and the five Subsamples*

|  |  | Total | ADOPT | Self-help comparison study | Enhancement | ESCAschool | WASH |
| --- | --- | --- | --- | --- | --- | --- | --- |
|  |  | NP | NP | NP | NP | NP | NP |
| PP | *r* | -.32 | -.34 | -.30 | -.39 | -.26 | -.23 |
|  | 95% CI | [-.36, -.28] | [-.40, -.27] | [-.47, -.12] | [-.53, -.22] | [-.33, -.18] | [-.32, -.14] |

*Note.* PP = Positive Parenting Scale. NP = Negative Parenting Scale. *N*_total_ = 1879. *n*_ADOPT_ = 695. *n*_Self-help comparison_ = 104.

*n*_Enhancement_ = 11. *n*_ESCA_ = 559. *n*_WASH_ = 407.

**References**

1. Döpfner M, Katzmann J, Hanisch C, Fegert JM, Kölch M, Ritschel A, et al. Affective

dysregulation in childhood - optimizing prevention and treatment: protocol of three randomized

controlled trials in the ADOPT study. BMC Psychiatry. 2019;19(1):264.

2. Hautmann C, Dose C, Duda-Kirchhof K, Greimel L, Hellmich M, Imort S, Katzmann J, Pinior

J, Scholz K, Schürmann S, Wolff Metternich-Kaizman T, Döpfner M. Behavioral versus

nonbehavioral guided self-help for parents of children with externalizing disorders in a randomized

controlled trial. Behav Ther. 2018;49(6):951-965.

3. Dose C, Hautmann C, Bürger M, Schürmann S, Woitecki K, Döpfner M. Telephone‐assisted

self‐help for parents of children with attention‐deficit/hyperactivity disorder who have residual

functional impairment despite methylphenidate treatment: A randomized controlled trial. J Child

Psychol Psychiatry. 2017;58(6):682-690.

4. Döpfner M, Hautmann C, Dose C, Banaschewski T, Becker K, Brandeis D, et al. ESCAschool

study: trial protocol of an adaptive treatment approach for school-age children with ADHD including

two randomised trials. BMC Psychiatry. 2017;17(1):1-14.

5. Döpfner M, Plück J. Evaluation eines web-assistierten Selbsthilfe-Trainings für Eltern von

Kindern mit Aufmerksamkeitsdefizit- / Hyperaktivitätsstörung (WASH). In Amelung VE, Eble S,

Hildebrandt H, Knieps F, Lägel R, Ozegowski S, Schlenker RU, Sjuts R (Eds.), Innovationsfonds.

Impulse für das deutsche Gesundheitssystem (pp. 362-367). MWV Medizinisch Wissenschaftliche

Verlagsgesellschaft; 2017.

6. Canadian Attention Deﬁcit Hyperactivity Disorder Resource Alliance. Canadian ADHD

practice guidelines (3rdedn). Toronto, ON: Author; 2011.

7. Treier AK, Döpfner M, Ravens-Sieberer U, Görtz-Dorten A, Boecker M, Goldbeck C, et al.

Screening for affective dysregulation in school-aged children: relationship with comprehensive

measures of affective dysregulation and related mental disorders. Eur Child Adolesc Psychiatry.

2024;33(2):381-390.

8. Hurley KD, Huscroft-D’Angelo J, Trout A, Griffith A, Epstein M. Assessing parenting skills

and attitudes: A review of the psychometrics of parenting measures. J Child Fam Stud.

2014;23(5):812-823.

9. Parent J, Forehand R. The Multidimensional Assessment of Parenting Scale (MAPS):

Development and psychometric properties. J Child Fam Stud. 2017;26(8):2136-2151.

10. Shelton K, Frick P, Wootton J. Assessment of parenting practices in families of elementary

school-age children. J Clin Child Psychol. 1996;25(3):317-329.

11. Robinson C, Mandleco B, Olsen SF, Hart CH. Authoritative, authoritarian, and permissive

parenting practices: Development of a new measure. Psychol Rep. 1995;77:819-830.

12. Arnold D, O’Leary S, Wolff L, Acker M. The Parenting Scale: A measure of dysfunctional

parenting in discipline situations. Psychological Assessment. 1993;5(2):137-144.

13. Perepletchikova F, Kazdin AE. Assessment of parenting practices related to conduct

problems: Development and validation of the Management of Children's Behavior Scale. J Child Fam

Stud. 2004;13(4):385-403.

14. Schaefer ES. Children's reports of parental behavior: An inventory. Child Dev.

1965;36(2):413-424.

15. Lovejoy MC, Weis R, O'Hare E, Rubin EC. Development and initial validation of the Parent

Behavior Inventory. Psychol Assess. 1999;11(4):534-545.

16. McEachern AD, Dishion TJ, Weaver CM, Shaw DS, Wilson MN, Gardner F. Parenting

Young Children (PARYC): Validation of a self-report parenting measure. J Child Fam Stud.

2012;21(3):498-511.

17. Stattin H, Kerr M. Parental monitoring: A reinterpretation. Child Dev. 2000;71(4):1072-1085.

18. Gerard AB. Parent-child relationship inventory (PCRI). Western psychological services;

1994.

19. Milner JS. The Child Abuse Potential (CAP) inventory. John Wiley & Sons, Inc; 2004.

20. Milner JS. Assessing physical child abuse risk: The child abuse potential inventory. Clin

Psychol Rev. 1994;14(6):547-583.

21. Milner JS, Gold RG, Wimberley RC. Prediction and explanation of child abuse: Cross-

validation of the Child Abuse Potential Inventory. J Consult Clin Psychol. 1986;54(6):865-866.

22. Abidin RR, Konold TR. PAM: Parenting alliance measure: Professional manual.

Psychological Assessment Resources; 1999.

23. Konold TR, Abidin RR. Parenting alliance: A multifactor perspective. Assessment.

2001;8(1):47-65.

24. DfES. “The Special Educational Needs (SEN) Code of Practice”. DfES; 2001.

25. Strayhorn JM, Weidmann CS. A parent practices scale and its relation to parent and child

mental health. J Am Acad Child Adolesc Psychiatry. 1988;27:613-618.

26. Hahlweg K, Bodenmann G. Universelle und indizierte Prävention von Beziehungsstörungen.

In Sozialpsychologie der Partnerschaft (pp. 191-217). Springer; 2003.

27. Kazdin AE, Rogers A. Management of Children’s behavior Scale. Pittsburg: Western

Psychiatric Institute and Clinic. University of Pittsburg School of Medicine; 1985.

28. Imort S, Hautmann C, Greimel L, Katzmann J, Pinior J, Scholz K, et al. Fragebogen zum

positiven und negativen Erziehungsverhalten (FPNE): Eine psychometrische Zwischenanalyse

[Questionnaire about positive and negative parenting behaviour (FPNE): A psychometric interim

analysis]. Poster presented at the 32. Symposium der Fachgruppe Klinische Psychologie und

Psychotherapie der DGPs, Bielefeld, Germany; 2014.
